# Survival rate of patients with bladder cancer and its related factors in Kurdistan Province (2013–2018): a population-based study

**DOI:** 10.1186/s12894-020-00769-1

**Published:** 2020-12-11

**Authors:** Mozhdeh Amiri, Sofimajidpour Heshmatollah, Nader Esmaeilnasab, Jamshid Khoubi, Ebrahim Ghaderi, Daem Roshani

**Affiliations:** 1 Department of Epidemiology and Biostatistics, Faculty of Medicine, Kurdistan University of Medical Sciences, Sanandaj, Iran; 2 Department of Urology, Faculty of Medicine, Kurdistan University of Medical Sciences, Sanandaj, Iran; 3 Social Determinants of Health Research Center, Research Institute for Health Development, Kurdistan University of Medical Sciences, Sanandaj, Iran; 4 Environmental Health Research Center, Research Institute for Health Development, Kurdistan University of Medical Sciences, Sanandaj, Iran

**Keywords:** Survival rate, Bladder cancer, Population based, Cox proportional hazards model

## Abstract

**Background:**

Bladder cancer is one of the most common urinary tract cancers. This study aims to estimate the survival rate of patients with bladder cancer according to the Cox proportional hazards model based on some key relevant variables.

**Methods:**

In this retrospective population-based cohort study that explores the survival of patients with bladder cancer and its related factors, we first collected demographic information and medical records of 321 patients with bladder cancer through in-person and telephone interviews. Then, in the analysis phase, Kaplan–Meier method and log-rank test were used to draw the survival curve, compare the groups, and explore the effect of risk factors on the patient survival rate using Cox proportional hazards model.

**Results:**

The median survival rate of patients was 63.2 (54.7–72) months and one, three and five-year survival rates were 87%, 68% and 54%, respectively. The results of multiple analyses using Cox's proportional hazards model revealed that variables of sex (male gender) (HR = 11.8, 95% CI: 0.4–100.7), more than 65 year of age (HR = 4.1, 95% CI: 0.4–11), occupation, income level, (HR = 0.4, 95% CI: 0.2–0.8), well differentiated tumor grade (HR = 3.2, 95% CI: 1.7–6) and disease stage influenced the survival rate of patients (*p* < 0.05).

**Conclusion:**

The survival rate of patients with bladder cancer in Kurdistan province is relatively low. Given the impact of the disease stage on the survival rate, adequate access to appropriate diagnostic and treatment services as well as planning for screening and early diagnosis, especially in men, can increase the survival rate of patients.

## Background

Bladder cancer is the most common urinary tract cancer and the ninth most prevalent cancer in both sexes worldwide [1, 2], accounting for 7% and 2% of new cases of cancers in men and women, respectively [3]. According to statistical center of Iran, the ratio of bladder cancer mortality in men was 3.26 times higher than that of women between 2006 and 2010. The incidence of bladder cancer mortality was 1.12 and 1.09 per 100,000 people in 2006 and 2010, respectively, indicating that the trend was relatively stable. According to a 2015 study by Mahdavi et al., the death rate of bladder cancer increases with age, so that people above 70 years of age are more vulnerable to this disease [4]. Currently, bladder cancer mortality in Europe and the United States is declining due to the lower prevalence of smoking [5]. In 2016, 76,960 new cases and 16,390 deaths related to bladder cancer were reported in the United States, of which 76% of new cases were reported in men [6]. Women accounted for fewer cases, but factors like late diagnosis and advanced stages of the disease decreased their survival rate [7–11]. Bladder cancer is the second and the third most common cancer among men in Markazi and Kurdistan province, respectively [12, 13]. Studies on bladder cancer have linked several factors, including occupation, smoking, and sex to the higher prevalence of the disease [11, 14, 15].

The survival rate index is defined as the proportion of cancer patients who survive over a given period of time after diagnosis. Several variables, such as cancer histology and stage, as well as the availability of treatment, affect the survival of the disease [18]. According to the results of our study, despite the rising disease incidence in Iran, there is a paucity of population-based research on the survival rate of patients with bladder cancer in Iran, and most of studies in this field have focused on the cancer trend and epidemiological analysis [12, 16–18].

Hence, the aim of the present study is to estimate the survival rate of bladder cancer patients based on Cox's proportional hazards model and to explore the variables affecting survival rates based on population-based information derived from Kurdistan province. The findings can significant contribute to the health system planning, timely treatment, patient survival and quality of life.

## Methods

The present study is a retrospective population-based cohort study conducted in Kurdistan Province from 2013 to 2018. In the first stage, the information on patients diagnosed with bladder cancer was collected by reviewing all cases recorded in cancer registry at the Provincial Health Department, the Cancer Registration Center and the Provincial Mortality Registration Center. In the next step, demographic information (including: gender, age at diagnosis, marital status, place of residence, occupation, level of education, social status, smoking, family history and exposure to toxins and pesticides) and medical and pathological information (including stage of disease, tumor differentiation grade, treatment method, histology, diagnosis method and comorbidity) were collected through interviews, questionnaires and the review of medical records.

Patient information was recorded based on the ICDL10 International Classification (codes C67.0-C67.9). Given that all diagnosed patients in the study were chosen over a 6-year period from 2013 to 2018, the research was undertaken as a population census in Kurdistan province. In this study, of 341 patients diagnosed with bladder cancer, 12 (3.5%) refused to participate in the study, and 8 (2.3%) could not be reached. Hence, finally, 321 subjects were including in the study and their information was recorded. The cause and date of patients' deaths were recorded based on inquiries from the family and death certificates. Patients who were lost to the follow-up or died of bladder cancer were excluded from the study.

### Statistical analysis

To determine the relationship between study variables and bladder cancer survival, STATA V.14 statistical software, Kaplan–Meier nonparametric method, and the log-rank test were used. For all variables, the survival curve was drawn and the variables with *p* values < 0.2 in the log-rank test were included in Cox proportional hazards model to estimate the effect of multiple variables on the survival rate by calculating the hazard ratio (HR) at a significant level of less than 0.05. To calculate the total survival rate and one, three and five-year survival rates, the life table (monthly) was used over 1 to 5- year period. Three methods of scaled Schoenfeld residuals, the hazard log cumulative and goodness of fit were used to assess the appropriateness of the hazards.

## Results

**Fig. 1:**
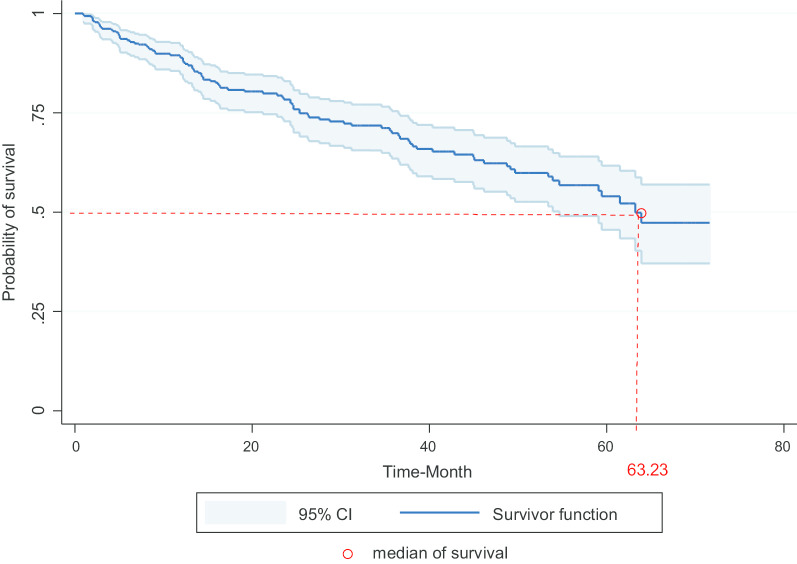
Survival rate of patients treated with bladder cancer in Kurdistan province (2013–2018) (Kaplan–Meier). CI = confidence interval, BC = bladder cancer

In this study, the survival rate analysis was conducted for 321 patients with bladder cancer, of which 82.2% were male and 17.7% were female. Also, 77% of patients lived in urban areas, 87.8% were married and 53.9% were above 65 years of age. During the study period, 96 out of 321 participants passed away, 86 due to bladder cancer and 10 due to other causes. According to the results, the overall median survival of patients was 63.2(54.7–72) months and the mean survival (47.1–53.7) was 50.4 ± 1.7 months. Also, the one to five-year survival rates were 87%, 76%, 68%, 61% and 54%, respectively (Fig. 1).

Table 1 shows one-year, two-year, three-year and five-year survival rates for each variable using the life table, as well as *p* values obtained from the log-rank test.

**Table 1:** Demographic and clinical characteristics of patients diagnosed with bladder cancer in terms of mean survival rate using Kaplan–Meier method

Characteristic	Category	Frequency (%)	Mean survival per month (95% CI)	p*
Sex	Female	57 (17.7)	57.4 (50–64.8)	0.04
	male	264 (82.2)	48.9 (45.2–52.6)	
Age at diagnosis	≤ 50 y	43 (13.4)	60.3 (53.1–67.5)	< 0.001
	51–64 y	105 (32.7)	55.8 (50.5–61)	
	≥ 65 y	173 (53.9)	44.3 (39.7–48.9)	
Marital status	Single (unmarried, divorced, widow/widower)	39 (12.1)	32.7 (23.8–41.5)	< 0.001
	Married	282 (87.8)	52.9 (49.5–56.4)	
Residence	Rural	74 (23.5)	41.7 (35.3–47.9)	< 0.001
	Urban	247 (76.9)	53.9 (50.2–57.7)	
Occupation	Unemployed/Retired	67 (20.8)	58.9 (52.5–65.3)	0.008
	Housewife	47 (14.6)	56.2 (47.9–64.5)	
	Worker	53 (16.5)	48.8 (39.6–57.9)	
	Self-employed	66 (20.5)	48.1 (40.5–55.7)	
	Office job	27 (8.4)	47.7 (39.3–55.9)	
	Agriculturist	61 (19.0)	41.6 (34.9–48.2)	
Education	Illiterate	158 (49.2)	45.3 (40.5–50.1)	0.001
	Literate	163 (50.7)	55.8 (51.4–60.2)	
Socioeconomic status	Poor	106 (33.1)	42.4 (36.9–47.8)	< 0.001
	Moderate	107 (33.4)	52.3 (46.5–57.9)	
	Rich	107 (33.4)	58.5 (53.2–63.8)	
Smoking	No	172 (53.5)	51.7 (46.6–56.7)	0.5
	Yes	149 (46.4)	49.4 (45–53.7)	
Family history of BC	No	301 (93.7)	50.0 (46.5–53.4)	0.5
	Yes	20 (6.2)	54.6 (43.3–65.9)	
Comorbidity	No	205 (63.8)	50.8 (46.7–54.8)	0.5
	Yes	116 (34.1)	49.3 (43.6–54.9)	
TNM stage	I	210 (65.4)	58.5 (55–62)	< 0.001
	II	74 (23)	44.5 (37.2–51.7)	
	III	29 (9)	21.7 (12.9–30.4)	
	IV	8 (2.5)	21.3 (8.9–33.6)	
Grade	Poorly differentiated	217 (67.6)	59.7 (56.3–62.9)	< 0.001
	Well differentiated	104 (32.4)	31.6 (25.8–37.3)	
Treatment	Radiation therapy	13 (4)	48.2 (34.5–61.7)	0.05
	Surgery	207 (64.5)	53.6 (49.6–57.5)	
	Chemotherapy	10 (3.1)	31.9 (20.3–43.5)	
	Combinational treatment (Surgery, Radiotherapy, Chemotherapy or Immunotherapy)	85 (26.4)	44.5 (38–50.8)	
Histology	Adenocarcinoma	10 (3.1)	34.7 (24.8–44.5)	0.03
	Squamous cell carcinoma	7 (2.1)	24.9 (3.7–46)	
	Urothelial carcinoma	304 (94.7)	51.3 (47.9–54.7)	
Method of diagnosis	Urinalysis and ultrasound	55 (17.1)	52.9 (46.5–59.3)	0.5
	Cystoscopy and biopsy	55 (17.1)	45.4 (37.7–53)	
	Biopsy and ultrasound	35 (10.9)	50.0 (40.6–59.3)	
	Combinational method of diagnosis	176 (54.8)	50.8 (46.1–55.6)	
Poison and pesticides exposure	Yes	64 (19.9)	49.9 (43.5–56.4)	0.9
	No	257 (80)	50.5 (46.7–54.3)	

*BC* bladder cancer

*Log-rank test-*p* value

According to Table 1, the survival rate was higher in women than in men, and according to the log-rank test, the survival curve was not significantly different between the subgroups (*p* < 0.05). Moreover, survival rates were lower in single patients, patients above 65 years of age, and patients living in rural areas. Survival rates in retired and unemployed patients were higher than other groups. The literate and high-income patients also had higher survival rates. In this study, smoking, supplementary insurance, and family history were not statistically significant.

As shown in Table 1, patients at early disease stage who did not have an underlying disease, had a poorly differentiated tumor grade, and their histology was urothelial carcinoma had a higher survival rate. However, the survival rate was not significantly different with regard to the method of diagnosis, type of treatment and exposure to poisons and pesticides. According to the results, group curves, gender (*p* = 0.044), age at diagnosis (*p* < 0.001), residence (*p* < 0.001), marital status (*p* < 0.001), level of education (*p* = 0.002), occupation (*p* = 0.008), socioeconomic status (*p* < 0.001), disease stage (*p* < 0.001), histology (*p* = 0.04) and tumor grade differentiation (*p* < 0.001) were significantly different. Moreover, the curves of the supplementary insurance, smoking, method of diagnosis, family history and underlying disease were not significantly different patients (*p* < 0.05) (Table 1).

In the multivariate analysis, using Cox's proportional hazards, variables of gender, age groups, residence, marital status, level of education, occupation, disease stage, histology, treatment method, differentiation grade, and socioeconomic status were incorporated in the final model of Cox's proportional hazards model (*p* < 0.2). The results of the final model revealed that the hazard ratio was significantly higher in men than in women (HR = 11.8, 95% CI: 0.4–100.7). Moreover, the hazard ratio increased with age (HR = 4.1, 95% CI: 1.5–11.1) and patients from a high socioeconomic background had a lower hazard ratio than those from a poor socioeconomic background (HR = 0.4, 95% CI: 0.2–0.8). The hazard ratio of patients at Stage II (HR = 1.5, 95% CI: 0.7–2.9), Stage III (HR = 2.8, 95% CI: 1.34–5.8) and Stage IV (HR = 6.4, 95% CI: 2.1–19.2) was higher than Stage V. Also, the hazard ratio growth at Stages III and IV was significantly different than Stage I, but it was not the case for Stages I and II (*p* < 0.05).

Moreover, the hazard ratio at well differentiated grade was greater than the poorly differentiated tumor grade (HR = 3.25, 95% CI: 1.7–6). Patients with an office job (HR = 4.9, 95% CI: 1.7–13.9) and workers (HR = 2.6, 95% CI: 1.1–6.2) had a higher hazard ratio than retired and unemployed patients. Meanwhile, the variables of residence, marital status, level of education, type of histology and treatment method were not significantly related to the survival rate of patients (*p* < 0.05) (Table 2).

**Table 2:** Univariate and multivariate Cox proportional hazards model for the overall survival of patients with bladder cancer

	Univariate model		Multivariate model	
	HR (95%)	*p* value	HR (95%)	*p* value
Sex				
Female	1	–	1	–
Male	1.9 (1–3.7)	0.04	11.8 (1.3–100.7)	0.02
Age				
≤ 50	1	–	1	
51–64	1.6 (0.6–4)	0.27	1.1 (0.4–3.1)	0.8
≥ 65	3.3 (1.4–7.5)	0.005	4.1 (1.5–11)	0.004
Marital status				
Single*	1	–	1	–
Married	0.36 (0.2–0.6)	< 0.001	0.7 (0.3–1.3)	0.3
Residence				
Rural	1	–	1	–
Urban	0.5 (0.3–0.7)	0.001	0.8 (0.4–1.6)	0.5
Occupation				
Unemployed/Retired	1	–	1	–
Housewife	1.2 (0.5–26.8)	0.6	11.3 (1.1–116.8)	0.04
Worker	2.2 (1–4.7)	0.04	2.6 (1.1–6.2)	0.03
Self-employed	2.2 (1.1–4.4)	0.03	3.3 (1.5–7.1)	0.002
Office job	1.8 (0.7–4.4)	0.19	4.9 (1.7–13.9)	0.003
Agriculturist	3.1 (1.6–6.1)	0.001	1.8 (0.7–4.4)	0.2
Education				
Illiterate	1	–	1	–
Literate	0.5 (0.3–0.8)	0.003	1.1 (0.6–2)	0.8
Socioeconomic status				
Poor	1	–	1	–
Moderate	0.6 (0.3–0.9)	0.02	0.5 (0.3–1)	0.06
Rich	0.35 (0.2–0.6)	< 0.001	0.4 (0.2–0.8)	0.01
TNM stage				
I	1	–	1	–
II	2.4 (1.5–4)	< 0.001	1.5 (0.7–2.9)	0.2
III	7.9 (4.7–13.4)	< 0.001	2.8 (1.3–5.8)	0.006
IV	7.3 (3.2–16.6)	< 0.001	6.4 (2.1–19.3)	0.001
Grade				
Poorly differentiated	1	–	1	–
Well differentiated	5.2 (3.4–7.9)	< 0.001	3.2 (1.7–6)	< 0.001
Treatment				
Radiation therapy	1	–	1	–
Surgery	0.8 (0.3–2.1)	0.6	0.9 (0.3–2.6)	0.8
Chemotherapy	1.7 (0.4–6.6)	0.4	1.5 (0.3–6.2)	0.6
Combinational treatment	1.3 (0.4–3.7)	0.6	0.9 (0.5–5)	0.4
Histologic type				
Adenocarcinoma	1	–	1	–
Squamous cell carcinoma	1.3 (0.3–5.1)	0.7	4.7 (0.8–26.8)	0.08
Urothelial carcinoma	0.46 (0.19–1)	0.06	1.6 (0.5–4.5)	0.4

*HR* hazard ratio, *CI* confidence interval

*Unmarried, divorced, widow/widower

Figure 1 shows that the median survival of patients with bladder cancer in Kurdistan province was 63.2(54.7–72) months.

**Fig. 2:**
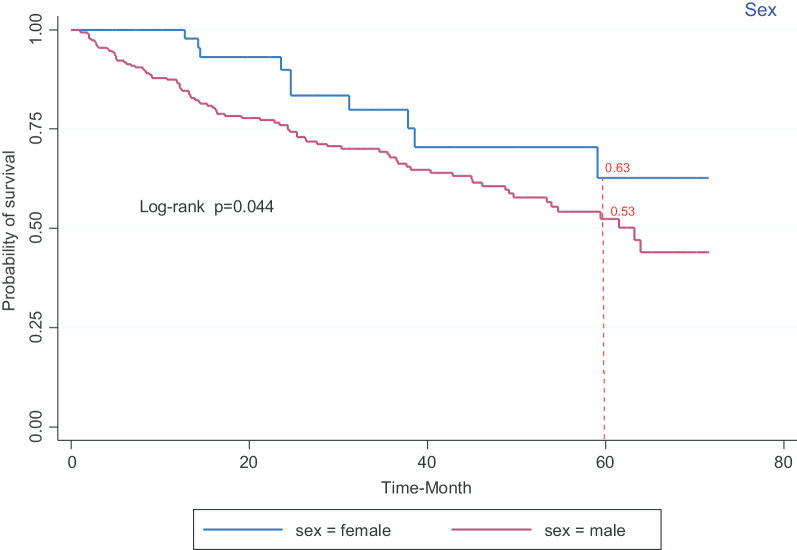
Kaplan–Meier curves of survival in patients with bladder cancer versus sex

**Fig. 3:**
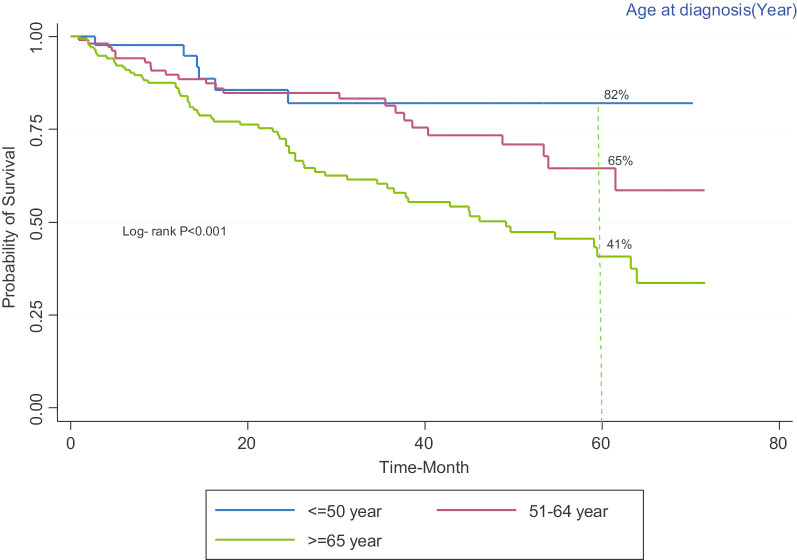
Kaplan–Meier curves of the survival of patients with bladder cancer versus age at diagnosis

The survival rate of patients with bladder cancer versus sex in study participants shows that the five-year survival is higher in women than in men (Fig. 2). Also, the survival rate of patients with bladder cancer versus age at diagnosis shows that the five-year survival in participants with ≥ 65 year is less than the others (Fig. 3).

**Fig. 4:**
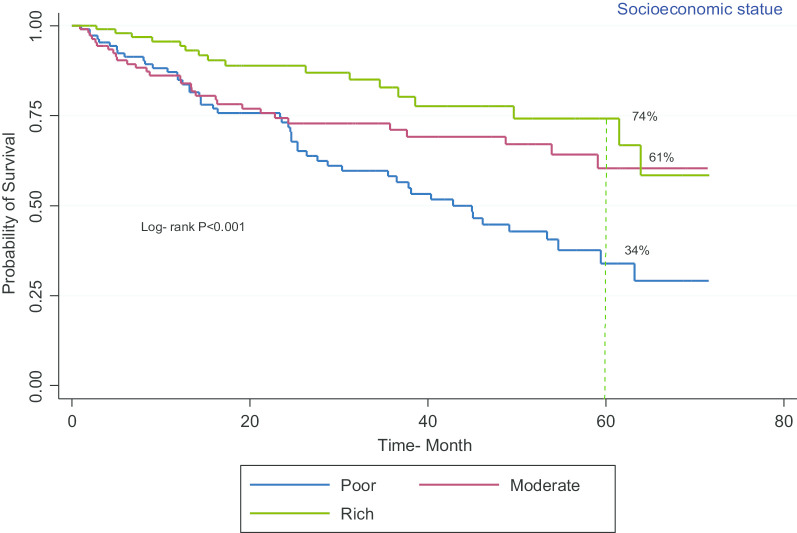
Kaplan–Meier curves of the survival of patients with bladder cancer versus socioeconomic status

Figure 4 shows that the five-year survival rate in patients from rich (74%), moderate (61%), and poor (34%) socioeconomic backgrounds, according to the log-rank test, is significantly different (*p* < 0.001).

**Fig. 5:**
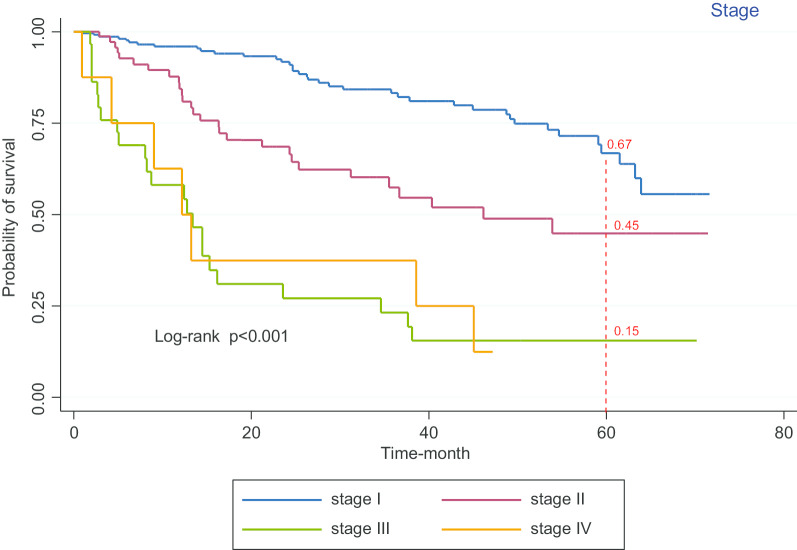
Kaplan–Meier curves of the survival of patients with bladder cancer versus diagnosis stage

Figure 5 shows the five-year survival rate in patients at Stage I (67%), Stage II (45%) and Stage III (15%), according to the log-rank test, is not significantly different (*p* < 0.001).

## Discussion

The one-year, three-year and five-year survival rates of patients with bladder cancer in Kurdistan province resembled the survival rates of patients in Shiraz province, as reported by Mohammad Beigi et al. The one-year, three-year, five-year, and ten-year survival rates of patients in their study were 0.9, 0.7, 0.6, and 0.2, respectively [19].

According to the American Cancer Society (ACS) in 2016, the five-year, ten-year, and fifteen-year survival rates at all stages of bladder cancer are 77%, 70%, and 65%, respectively [20]. This suggests that the five-year survival rate of bladder cancer patients in Kurdistan province (54%) in this study is lower than in developed countries. However, the median survival of patients was estimated to be 63.23 months, which is higher than the value reported by Benjamin WF et al. (Median OS = 14, 95% CI: 13.5–14.5) [21]. It is probably due to early diagnosis of the disease because 65.4% of patients were identified at Stage I and 23.05% at Stage II.

In this study, the history of smoking was not significantly related to the survival rate of patients. Most patients had a history of smoking at young age but had abandoned smoking after a while or due to the bladder cancer. This could be attributed to the effect of smoking cessation followed by the reduced devastating effect of the disease. Our findings are consistent with those reported by Mohammad Beigi et al., who did not report a significant relationship between smoking and survival rates [19]. This results illustrated that just as reduced smoking decreased the incidence of bladder cancer, smoking cessation also increased the survival of patients with bladder cancer [22].

In the present study, age and income level influenced the survival rate of patients with bladder cancer (*p* < 0.05), which is aligned with the study of Joshua Lara et al. In their study, the risk of death in adolescents and youths was lower than older people (HR = 0.4, 95% CI: 0.3–0.5). In adolescents and young people from poor socioeconomic background, the specific survival rate (HR = 7.1, *p* < 0.001) and the overall survival rate (HR = 5, *p* < 0.001) were statistically significant [23].

According to the results, the survival rate was higher in women than in men, which is in conflict with the findings reported by Stephen B.W. et al. This could be assigned to the early detection of the disease and the higher commitment of women with bladder cancer to follow-ups. However, in both studies, the incidence was higher in men. Furthermore, old age, tumor stage and single marital status were found to be significantly related to the lower survival rate (*p* < 0.05). In this study, we observed that the diagnosis of the disease at lower stage was associated with the higher survival of patients, which is consistent with the study of Stephen B.W. [24].

Survival rates were also higher in urban residents than in rural areas, which can be attributed to the greater access to healthcare services and follow-ups. For many cancers, survival is considerably lower in rural areas than in urban areas (*p* < 0.05) [25].

Khoubi et al. introduced exposure to poisons and pesticides as a risk factor for bladder cancer, but in the present study, the history of exposure to pesticides was not significantly correlated with the survival rate of patients with bladder cancer (*p* > 0.05) [26]. We found that most farmers had attended Agricultural Jihad’s training classes and put on personal protective gears during spraying. There was a statistically significant association between housework and increased risk of bladder cancer in women, which is in agreement with the findings of Khoubi et al. (ISCO 5152, OR = 5.9, 95% CI = 1.04–34.3) [26]. This is due to the fact that women are more likely to use detergents, cleaners and bleaches, and inhale oil fumes during frying and less likely to engage in physical activities. Our study also showed that an office job and self-employment were significantly correlated with the survival rate (*p* < 0.05).

The univariate and multivariate Cox regression analysis did not reveal a significant relationship between treatment method and survival rate, but in the study of Mark C. et al., the overall survival rate improved in chemotherapy patients (HR = 0.7, 95% CI: 0.6–0.9, *p* < 0.001) [27]. In this study, 64.9% of patients with bladder cancer underwent surgery and 3.1% started chemotherapy, but the surgical procedure was not significantly correlated with the patient survival (*p* < 0.05). This aligns well with the study of Stephen BW et al. who did not report any associations between the survival of the elderly with radical cystectomy and sex at all stages (HR = 1.07, 95% CI: 1.01–1.1, *p* = 0.02) [24].

Literacy was also linked to survival. Literate participants had a higher survival rate than the illiterate ones. This suggests that raising awareness and promoting health literacy can influence the survival rate of patients (*p* < 0.05). The survival rate was higher in married subjects than in the single subjects (never married, widower or divorced), which is in agreement with the study of Klapheke et al. [28].

Our study had a number of limitations including incompleteness of patients' medical records. The distinguishing point of this study is its population-based nature, which eliminates the problem of accessing hospital records and biases.

## Conclusion

The results of this study demonstrated that the survival rate of patients with bladder cancer in Kurdistan province is relatively low due to lack of access to appropriate diagnostic and treatment services, lack of screening and early diagnosis and sloppy follow-ups, especially amongst men. Variables of gender, over 65 years of age, occupation, socioeconomic background, tumor differentiation grade, and disease stage affected patient survival rates. This suggests the importance of further planning for these factors to increase the survival of patients with bladder cancer.

## Data Availability

The datasets used during the study could be provided by the corresponding author on reasonable request.
